# Diet and non-Hodgkin's lymphoma risk

**Published:** 2012-06-28

**Authors:** Zahra Mozaheb, Amir Aledavood, Farzaneh Farzad

**Affiliations:** 1 Department of Hematology, Mashhad University of Medical Science, Mashhad, Iran; 2 Department of Radiotherapy and Oncology, Mashhad University of Medical Science, Mashhad, Iran; 3 Azad University of Mashhad, Iran

**Keywords:** Non-Hodgkin lymphoma, diet, risk factor, etiology

## Abstract

**Background:**

The role of dietary factors in the epidemiology of Non-Hodgkin's lymphoma (NHL) remains largely undefined. Dietary habits may play a role in the etiology of NHL by influencing the immune system.

**Methods:**

Dietary patterns and the risk of NHL were analyzed in a case control study; including 170 NHL cases and 190 controls. All subjects completed a validated food-frequency questionnaire. The dietary pattern was investigated separately and in nine nutritional groups. Crosstab tables were used to estimate the odds ratios (OR), and P_trend_.

**Results:**

Consumption of highest versus lowest quartile of proteins (OR, 8.088 P_trend_=0.000), fats (OR, 6.17 P_trend_=0.000) and sweets (OR, 8.806 P_trend_=0.000) were associated with a significantly increased NHL risk. The inverse association was found for fresh fruits (OR, 0.117 P_trend_=0.000) and vegetables (OR, 0.461 P_trend_=0.010).

**Conclusion:**

An association between dietary intake and the risk of NHL is biologically plausible due to immunosuppressive effects of fat and animal proteins, and antioxidant properties of vegetables and fruits.

## Background

Non-Hodgkin lymphoma is the fifth most frequently diagnosed cancer. The incidence of non-Hodgkin lymphoma has risen by at least 100 percent over the past five decades especially in the west. This increase has involved most geographic area, all adult age groups, and both sexes [1–3].

Immune deficiency, both congenital and acquired, is one of the most important risk factors of non-Hodgkin lymphoma [4]. Carotinoids, vitamins C and E might protect against carcinogenesis by neutralizing reactive oxygen and reducing oxidative DNA damage and mutation, and enhancing immune responses [5]. Inadequacy of folate intake results in abnormal DNA metylation and synthesis, chromosome breaks, and disruption of DNA repair [6]. Normal DNA synthesis may be important in lymphocyte proliferation to response to foreign stimuli. Vitamin A influences growth and differentiation of various hematopoietic progenitor cells and increase immunity [7]. Fruit and vegetables are rich sources of carotinoids, vitamin C and E, folate, and dietary fiber and other nutrient that may inhibit carcinogenesis [8].

Meats cooked at high temperatures, such as in pan-frying or grilling methods, is a source of carcinogenic heterocyclic amines and polycyclic aromatic hydrocarbons [9]; previous studies have shown that red meat is associated with cancers at a variety of sites, including the colorectal, stomach, pancreas, breast, prostate, kidney and lymphoma. An association between the dietary fat or proteins and NHL is plausible; as both may cause deleterious effects on the immune system [10]. A case-control study was conducted to examine the effects of various food products on the risk of developing NHL or their protective effects against it.

## Methods

### Study population

From 2007 to 2009, a case-control study was conducted (170 cases and 190 controls) on NHL in Mashhad. Cases included 110 men and 60 women, with the median age of 53 years. They were between 14 and 86 years old at diagnosis, histologically confirmed NHL (Table 1). They had no previous diagnosis of cancer, and they were alive at the interview time. All patients with NHL where admitted to “Omid Hospital” and also they were enrolled in an outpatient clinic. “Omid Hospital” is the biggest referral cancer center in the east of Iran (covering more than four provinces). Most patients suffering from different kinds of malignancies are referred to this center; where all kinds of adult lymphoma are captured.

**Table 1:** Descriptive characteristic of Non-Hodgkin's lymphoma cases and controls

	Case	Control
**Gender**		
Male	110	114
Female	60	76
**Total**	**170**	**190**
**Age (years)**		
Minimum	14	15
Maximum	86	83
Mean	51	47
Median	53	48

The NHL cases were classified and graded according to the Working Formulation (low, intermediate, or high), and histological type (diffuse or follicular) and Revised European-American Lymphoma/World Health Organization classification [11]. The majority of cases were diffuse large B cell lymphoma (DLBCL) and the second most common NHL was chronic lymphocytic leukemia/small lymphocytic lymphoma (CLL/SLL) [12].

Controls were patients between 18 and 83 years old. They had been admitted for a wide spectrum of diseases to other general hospitals. Patients who were admitted for other malignant diseases, chronic disease, or hepatitis and those that might have had their habits changed, were excluded from the control group. In total, 190 controls were selected randomly and accepted to participate completing the dietary questionnaire. 60% (n=114) of studied controls were males and 40% (n=76) were females with the median age of 48 years (Table 1). The exclusion criteria for the case group were HIV and HTLV1 positive patients.

Informed consent was obtained from all study participants before the study began according to the recommendations of the Ethical Committee of the Mashhad University of Medical Science.

### Interviews

Every case and every control participated in the interview and completed the questionnaire. Trained interviewers administered a prepared questionnaire to the case and the control groups to assess their habitual diet during the two years preceding the interview.

A standardized, structured questionnaire “Food Frequency questionnaire” was designed by Block [13] and was used to assess the usual diet before their diagnosis for cases, and before the admission for the control group, ensuring that cases reported the preceding diet. The questionnaire including 60 items about food, they were modified to fit Iranian dietary habits, and then they were used for our major analysis. Food items were divided into nine groups; 1: dairy products including milk, cheese, yogurt and ice cream 2: carbohydrate products including bread, rice, macaroni, potato 3: fruits including all kinds of fruits and juices 4: vegetables including raw, cooked and root vegetables 5: cereals 6: proteins including egg, hamburger, sausages, red and white meat 7: fats including butter, cream, margarine, mayonnaise, full fat milk, fatty meat 8: sweets, desserts and jams 9: soft drinks. The participants were asked how often they consumed any of the above-mentioned items by choosing one of nine possible responses, the lowest intake was nothing and the highest intake was every week. The serving size was defined in common units (e.g. 1 teaspoon of sugar, 1 glass of milk). Frequency of food intake and serving size were multiplied to get number of servings.

### Data analysis

In the analysis, the individual food items separately, and in nine major food groups were used to assess the association between the risk of NHL and intakes of various food items and groups. Odds Ratios (OR) and corresponding 95% confidence intervals (CI) by the lowest (Q1) and highest (q4) quartile intake for risk of NHL were calculated by crosstabs and P_trend_ was computed by Chi-Square Tests. OR more than one is considered a risk factor and less than one a protective factor.

## Results

Table 1 shows the mean age of case and control groups. Cases and controls were almost the same in number and age at diagnosis as the result of frequency matching. The mean age of 170 cases was 51 years (SD=18), and the mean age of the 190 controls was 47(SD=14), the male/female ratio in the case group was 1.8 and in the control group 1.5.

Table 2 illustrate the mean serving of each group of major food products (9 item) including dairy, carbohydrates, fresh fruits, vegetables, cereals, proteins, fats, sweets and soft drinks, and the standard deviation.

**Table 2:** Mean and standard deviation of food group consumption in case and control groups

Food product	group	N	Mean	Std. Deviation
**Dairy**	**Case**	**170**	**7.3663**	**2099431**
	Control	190	7.7965	3.06019
**Carbohydrates**	**Case**	**170**	**11.7096**	**3.55943**
	Control	190	10.8402	3.10166
**Fresh fruits**	**Case**	**170**	**8.3972**	**3.36506**
	Control	190	11.6882	2.98302
**Vegetables**	**Case**	**170**	**8.7116**	**4.11155**
	Control	190	9.5188	4.06817
**Cereals**	**Case**	**170**	**10.3158**	**4.23357**
	Control	190	9.6882	3.85303
**Proteins**	**Case**	**170**	**6.7121**	**2.43021**
	Control	190	4.4443	3.61936
**Fats**	**Case**	**170**	**7.1926**	**3.43650**
	Control	190	4.6200	3.78319
**sweets**	**Case**	**170**	**8.8592**	**3.56543**
	Control	190	6.0000	3.39344
**Soft drinks**	**Case**	**170**	**12.5221**	**5.23369**
	Control	190	13.1012	5.00635

Table 3 presents the association between intakes of major food products and the risk of non-Hodgkin lymphoma. It shows the OR and 95% CI and P_trend_ of NHL by the lowest (Q1=less than 25% serving) and the highest (Q4=more than 75% serving) quartile intake of 9 items of Food Groups. In this table we can see a significant increased risk for Non-Hodgkin lymphoma with the highest versus the lowest quartile intake of carbohydrate rich products (OR=1.865 CI, 1.03-3.37 P=0,038), protein rich products (OR=8.088 CI, 4.1-15.7 P_trend_=0.000), fats (OR=6.176 CI, 3.2-11.7, P_trend_=0.000) and sweets (OR=8.806 CI, 4.5-17.2, P_trend_=0.000). A decreasing risk and protective effect was found for vegetables (OR=0.461CI, 0.2-0.8 P_trend_=0.010), fresh fruits (OR=0.117 CI, 0.1-0.2 P_trend_=0.000), and soft drink (OR, 0.52 CI, 0.2-0.9, P_trend_=0.030). Dairy products and cereal were not significantly related to NHL risk.

**Table 3:** Odd ratios and 95% CIs for non-Hodgkin's lymphoma by categories of proteins and fats

Food item	Group	N0(Q1)	N0(Q4)	Q1 -Q4	OR(95% CI)	P trend
**egg**	case	38	49	5.000-12.00	1.482(0.832-2.637)	0.117
	control	54	47			
**hamburger**	case	19	120	0.00-6.00	18.947(9.981-35.967)	0.000
	control	87	29			
**sausage**	case	19	106	0.00-8.00	20.224(10.395-9.337)	0.000
	control	87	24			
**fried beef**	case	28	129	0.00-6.00	7.898(4.519-13.804)	0.000
	control	72	42			
**Grilled beef**	case	44	114	0.00-6.00	7.104(4.146-12.174)	0.000
	control	85	31			
**Boiled beef**	case	24	105	0.00-10.00	11.181(5.966-20.954)	0.000
	control	69	27			
**Fried mutton**	case	53	80	0.00-9.00	0.535(0.324-o.881)	0.014
	control	40	113			
**Grilled mutton**	case	41	94	0.00-8.00	12.282(6.232-24.204)	0.000
	control	75	14			
**Boiled mutton**	case	53	60	6.00-14.00	0.676(0.406-1.128)	0.134
	control	49	85			
**Fried chicken**	case	43	78	0.00-10.00	10.047(4.998-20.194)	0.000
	control	72	13			
**Grilled chicken**	case	35	87	0.00-12.00	12.221(5.865-25.469)	0.000
	control	56	12			
**Boiled chicken**	case	72	66	6.00-12.00	1.326(0.832-2.136)	0.246
	control	81	56			
**Tuna fish**	case	42	49	2.00-9.00	1.268(0.714-2.253)	0.418
	control	50	46			
**Fried fish**	case	36	80	0.00-10.00	4.615(2.505-8.505)	0.000
	control	54	26			
**Grilled fish**	case	29	137	0.00-4.00	6.178(3.693-10.335)	0.000
	control	12	155			
**Butter**	case	62	57	3.25-12.00	1.337(0.756-2.366)	0.197
	control	48	33			
**Margarine**	case	45	125	0.00-2.00	0.992(0.620-1.586)	0.534
	control	50	140			
**Mayonnaise**	case	28	90	0.00-10.00	18.138(8.925-6.861)	0.000
	control	79	14			

Intake of specific food items in proteins and fats groups and their risk for NHL are shown separately in Table 4. We examined egg as well as every and each individual type of meat with different cooking methods, including fried red meat (beef, mutton, sausage, and hamburger), grilled and boiled red meat (beef and mutton), fried, grilled and boiled chicken and fish. As we can see egg consumption was not associated with an increased risk of NHL (ORs=1.482 & P_trend_=0.117). Most types of meat with different cooking methods were positively associated with the risk of NHL including sausages (OR for highest versus lowest quartile of intake, 20.22 CI, 0.00-39.3 P_trend_=0.000), hamburger (OR, 18.94 CI, 9.9-35.9 P_trend_=0.000), among the red meats, fried beef had the most effect (OR, 7.89 CI, 4.5-13.8 P_trend_=0.000) and boiled mutton had the least (OR,0.676 CI, 0.40-1.12 P_trend_=0.134). For chicken and fish intakes, fried and grilled, were associated with the NHL risk (OR, 10.04, 12.22, 4.61, 6.17 P_trend_=0.000), while the boiled chicken (OR, 1.32 P_trend_=0.24) was not. This investigation shows that the cooking method is also important in risk of NHL. In fats group cream, mayonnaise, mutton fat and fatty milk were associated with an increased risk of lymphoma (P_trend_=0.000), but butter and margarine did not have a significant effect. Solid oil (saturated fat) was the most frequently used oil for 149 cases; however, 41 cases did not use it at all.140 control most frequently used oil was liquid oil (unsaturated fat) including sunflower, olive and colza oil; the difference was statistically significant(P_trend_=0.000).

**Table 4:** ORs and 95% CIs for non-Hodgkin's lymphoma by categories of proteins and fats

Food item	Group	N0(Q1)	N0(Q4)	Q1 -Q4	OR(95% CI)	P trend
**egg**	case	38	49	5.000-12.00	1.482(0.832-2.637)	0.117
	control	54	47			
**hamburger**	case	19	120	0.00-6.00	18.947(9.981-35.967)	0.000
	control	87	29			
**sausage**	case	19	106	0.00-8.00	20.224(10.395-9.337)	0.000
	control	87	24			
**fried beef**	case	28	129	0.00-6.00	7.898(4.519-13.804)	0.000
	control	72	42			
**Grilled beef**	case	44	114	0.00-6.00	7.104(4.146-12.174)	0.000
	control	85	31			
**Boiled beef**	case	24	105	0.00-10.00	11.181(5.966-20.954)	0.000
	control	69	27			
**Fried mutton**	case	53	80	0.00-9.00	0.535(0.324-o.881)	0.014
	control	40	113			
**Grilled mutton**	case	41	94	0.00-8.00	12.282(6.232-24.204)	0.000
	control	75	14			
**Boiled mutton**	case	53	60	6.00-14.00	0.676(0.406-1.128)	0.134
	control	49	85			
**Fried chicken**	case	43	78	0.00-10.00	10.047(4.998-20.194)	0.000
	control	72	13			
**Grilled chicken**	case	35	87	0.00-12.00	12.221(5.865-25.469)	0.000
	control	56	12			
**Boiled chicken**	case	72	66	6.00-12.00	1.326(0.832-2.136)	0.246
	control	81	56			
**Tuna fish**	case	42	49	2.00-9.00	1.268(0.714-2.253)	0.418
	control	50	46			
**Fried fish**	case	36	80	0.00-10.00	4.615(2.505-8.505)	0.000
	control	54	26			
**Grilled fish**	case	29	137	0.00-4.00	6.178(3.693-10.335)	0.000
	control	12	155			
**Butter**	case	62	57	3.25-12.00	1.337(0.756-2.366)	0.197
	control	48	33			
**Margarine**	case	45	125	0.00-2.00	0.992(0.620-1.586)	0.534
	control	50	140			
**Mayonnaise**	case	28	90	0.00-10.00	18.138(8.925-6.861)	0.000
	control	79	14			

The result of fruits and carbohydrate analysis is presented separately in Table 5. Fruits include: a) citrus, b) apple, pear, and peach, c) melon and water melon, d) grapes and plums. Most fresh fruits except berries had a protective effect on NHL (OR, 0.068, 0.122, 0.126, 0.440 P_trend_=0.000). Other fruits products such as compote, natural juice and commercial juice were associated with an increased risk of lymphoma (OR, 7.144, 54.588, 21.39 P_trend_=0.000). Green and other vegetable were protective (OR, 0.282, 0.197, P_trend_=0.000), nevertheless, root vegetables were not (OR, 2.468 P_trend_=0.001).

**Table 5:** ORs and 95% CIs for non-Hodgkin's lymphoma by categories of fruits and vegetables

Food item	Group	NO(Q1)	NO(Q4)	Q1-Q4	OR(95%C I)	P trend
**Citrus**	case	87	25	8.00-18.00	0.068(0.037-0.125)	0.000
	control	27	114			
**Apple, pear, peach**	case	69	32	6.00-17.50	0.122(0.064-0.230)	0.000
	control	21	80			
**Melon & water melon**	case	85	36	6.00-18.00	0.126(0.067-0.238)	0.000
	control	20	67			
**Grapes, plums**	case	78	44	4.00-12.00	0.440(0.252-0.769)	0.000
	control	39	50			
**Root vegetable**	case	44	61	6.00-14.00	2.468(1.432-4.255)	0.001
	control	73	41			
**Green vegetable**	case	67	47	6.00-15.00	0.282(0.161-0.494)	0.000
	control	31	77			
**Other vegetable**	case	69	29	5.00-15.00	0.197(107-363)	0.000
	control	31	66			
**Compote**	case	43	96	0.00-6.00	7.144(4.019-12.701)	0.000
	control	80	25			
**Natural juice**	case	10	116	0.00-9.75	54.588(23.77-125.36)	0.000
	control	80	17			
**Commercial juice**	case	20	75	0.00-8.00	21.397(10.485-43.667)	0.000
	control	97	17			

Table VI shows the effect of separate item of dairy, carbohydrates and sweets on NHL. Among dairy products yoghurt (OR, 0.319, P_trend_=0.000) and buttermilk (OR, 0.494, P_trend_=0.008) had significant protective effects on NHL. Milk consumption did not have significant effect on NHL (OR, 0.724 P_trend_=0.130), but different kind of milk had different effect, low fat milk was protective (OR, 0.04, P_trend_=0.000) but fatty milk (OR, 23.01, P_trend_=0.000) was a risk factor. For carbohydrate group, traditional bread (OR, 0.423 P_trend_=0.002) and rice (OR, 0.551 P_trend_=0.016) taking were associated with a decreased risk of NHL, however, hamburger bread (OR, 16.7 P_trend_=0.000) and macaroni (OR, 3.223 P_trend_=0.000) were associated with an increased risk of NHL. Sweet and biscuit (OR, 4.724 P_trend_=0.000) jam and jelly (OR, 2.547 P_trend_=0.000) consumption were positively associated with the NHL risk.

## Discussion

One approach for a better understanding of carcinogenesis is to modulate mutation rates with both genetic and environmental factors. Mice that were deficient in DNA mismatch repair (MMR) due to homozygosity for a null allele of Mlh1 develop normally but are prone to lymphomas, intestinal adenomas and carcinomas, and skin gland tumors [14], on the other hand antioxidant-related nutrients can enhance DNA repair activity by modifying gene expression distinct from their direct antioxidant properties and are protective for lymphoma [15]. In this study we aimed to show the effect of the most important environmental factor (diet) on NHL.

Diet is an important risk factor for many cancers. High fat/low calcium diets are associated with increased tumorigeneses, whereas caloric restriction reproducibly increases lifespan and decreases tumors. Excessive consumption of protein can result in immune unresponsiveness as a result of chronic hyperstimulation. In animal models, dietary fat suppresses the immune system [16].

Patients who were HIV or HTLV1 positive, or received immunosuppressive therapy before they were diagnosed with lymphoma, were excluded from the study. Using the dietary intake during years before beginning of disease instead of the most recent diet helped to reduce bias.

The limitations of this study included: We could not measure the exact amounts of intakes. Some questions were not answered, and the moderate(no consume low or high) consumption of some food items led to smaller population analysis.

In this case and control study we found an increased risk of NHL with highest quartile intakes of protein rich products, particularly for fried and grilled meat, processed meat( hamburger, sausage), fats, and sweets, and a decreasing risk was found for fresh fruits, vegetables, and soft drinks.

One of the major findings from our study was an increased risk of NHL associated with a higher consumption of proteins and fats. There are different results from previous studies which focus on fat and protein intakes as risk factors for NHL. A large case and control study has suggested that high consumption of fats, meat, and dairy products may increase the lymphoma risk [17]. Erber E et al [18] in their study showed that fat and meat consumption was associated with a fivefold higher risk of follicular lymphoma in men (P_trend_ = 0.03). The other population-based case–control study suggests that meat consumption, well-done or not, does not increase the risk of NHL, meanwhile fat intake was associated with a significantly increased risk for NHL [10]. A previous cohort study [19] and a case–control study [20], similar to our study, found an increased risk for NHL in the highest tertile of red meat consumption, the cohort study identified that the main component contributing to this risk was hamburgers. The large case and control study showed an increased risk of NHL with high intakes of processed meat, cheese, eggs, and dessert foods and a positive associations with NHL were also found for high consumption of total fat [21].

Not only food components may be associated with cancer risk, but also cooking methods can significantly increase cancer risk [22]. Our study found an association with red meat, processed meat and the risk of NHL. Processed meat (hamburger and susage), fried and grilled red meat were associated with the highest risk, but boiled chicken was associated with the least risk of NHL. Carcinogens and mutagens, such as heterocyclic amines can be generated during cooking red meat; it has been shown that these compounds can induce immunotoxicity and lymphoma [23].

Tongzhang. Z et al [24] reported a significantly increased risk of NHL associated with animal protein(OR, 1.7 95% CI 1.2,2.4) and saturated fat(OR,1.9 95%CI 1.1,2.3). In our study animal and saturated fat intakes were associated with an increased risk of lymphoma. An increased risk of NHL associated with a high fat diet may lead to altered immunocompetence, and immune system impairment by acting on the cyclogenase, lipoxygenas, or cytochrome P-450 pathways or directly on cell function through its effects on cell membrane structure and function. Alteration in dietary fat was found to alter membrane phospholipid fatty acid composition in a variety of cellular and subcellular membranes; such alteration in lymphocytes, could lead to impaired immune function [23, 24].

In this study all fresh fruits (including: a) citrus, b) apple, pear, peach, c) melon and water melon, e) grapes plums), green and other vegetables except root vegetable intakes were found to have a significantly inverse relationship with the risk of NHL, but compotes and juices were associated with an increased risk of NHL. Based on this result, we can say that the fiber of fruits and vegetables has an important role in protection against cancers.

Several studies support an inverse association between intakes of fruits and vegetables and the NHL risk. Particularly vegetables consumption may relate to the induction of apoptosis and the growth arrest in preneoplastic and neoplastic cells, two important actions of isothiocyanates that can be found in cruciferous vegetables [17]. Sauvaget et al [25] have examined a cohort of more than 36000 atomic-bomb survivors of Hiroshima and Nagasaki, an estimation of the radiation dose and the history of their diet were available. They evaluated the joint effect of radiation exposure and fruit and vegetable consumption on the risk of cancer death. They concluded that a regular intake of fruit or vegetables reduced this risk significantly in people exposed to radiation. Linda E Kelemen. Et al [15] in their study mention that higher intake of vegetable, lutein zeaxanthin, and zinc are associated with a lower NHL risk. Several other studies including Han, X et al [26], Talamini R. et al [27], Cross, AJ et al [28], Zheng, TZ et al [29], Thompson, C. A. et al [30] Chang, E. T.et al [31] found that high consumption of fruits and vegetables was associated with the reduced risk of NHL. Chiu BC et al [32] did not find an association between vegetables and lymphoma. Carrie A. et al [33] found 32% better overall survival for NHL patients who had a higher pre-diagnosis intake of fruits and vegetables.

Vegetables and fruits rich in antioxidant nutrients are hypothesized to be chemoprotective against many type of cancer, including lymphoma, by several mechanisms including reduction of reactive oxygen species responsible for oxidative DNA damage, regulation of cell survival and apoptosis pathway, and protection of immune responses [34]. In addition Franceschi et al [35] hypothesized that alimentary fibers may affect the dilution, absorption, and/or breakdown of fat and animal protein in the gut, either directly or indirectly by modifying the gut microflora.

In this study we found that the consumption of carbohydrate rich products (OR, 1.865 P_trend_=0.038) and sweets (OR, 8.806 P_trend_=0.000), were associated with an increased risk of NHL. Among carbohydrate products, traditional bread and rice were associated with a decreased risk but white bread and macaroni were associated with an increased risk (Table 6). Zheng et al [27] reported that white bread one of the major source of transunsaturated fat intake, was associated with an increased risk of NHL in their study.

**Table 6:** Odd ratios and 95% CIs for non-Hodgkin's lymphoma by categories of dairy, carbohydrates and sweets

Food item	Group	O(Q1)	(Q4)	Q1	Q4	OR(95%CI)	P trend
**Milk**	case	57	55	5.00		0.724(0.437-1.199)	0.130
	control	57	76		9.00		
**Yoghurt**	case	54	33	6.00		0.319(0.183-0.554)	0.000
	control	49	94		12.00		
**Cheese**	case	34	49	6.00		1.375(0.786-2.404)	0.165
	control	62	65		12.00		
**Ice cream**	case	60	80	2.00		1.052(0.648-1.707)	0.468
	control	56	71		6.00		
**Dough**	case	58	38	4.00		0.494(0.286-0.851)	0.008
	control	52	69		10.00		
**Traditional bread**	case	74	44	9.00		0.423(0.241-0.743)	0.002
	control	38	52		27.00		
**White bread**	case	29	100	0.00		16.749(8.959-31.312)	0.000
	control	102	21		8.00		
**Sweets and biscuit**	case	42	67	4.00		4.724(2.623-8.511)	0.000
	control	77	26		12.00		
**Jam, Jelly**	case	88	67	5.00		2.547(1.510-4.294)	0.000
	control	97	29		18.00		
**Rice**	case	70	44	12.00		0.551(0.328-0.926)	0.016
	control	57	65		21.00		
**Macaroni**	case	41	64	5.00		3.223(1.802-5.762)	0.000
	control	64	31		15.00		
**Pizza**	case	10	137	0.00		43.840(20.02-95.97)	0.000
	control	80	25		8.75		

Sweets group, including all kinds of sweets and biscuits, jams and jellies were associated with an increasing risk of NHL. It hypothesized that insulin and insulin like growth factor effect human cell growth particularly on lymphoid, erythroid and myeloid cells. In addition, insulin like growth factor has a proliferative effect on lymphoma and leukemic cells [25].The state of hyperglycemia is also known to initiate a reactive oxygen species chain reaction and to activate several proinflammatory cytokines, such as interleukin-6 and TNF-a, which have a role in NHL pathogenesis [36].

The other finding in our study was the relation of dairy product with the risk of NHL. Overall, milk was associated with a decreased risk. We found that low fat milk, yoghurt, and buttermilk, were associated with a decreased risk, but fatty milk and cheese were associated with an increased risk of NHL. Ross and Bras [37] have reported an increased risk of lymphoma in experimental rats after augmentation of the diet with casein, the major protein of milk. All positive findings would need to be confirmed in a different follow up study.

## Conclusion

Based on this study, it seems that dietary factors are associated with NHL risk; therefore, all people especially who are in high risk families would be well advised to pay particular attention to their diet. High beef consumption appears as a risk factor, while vegetables appear to be protective in cases with p53 over-expression [38]. The result of this study supports the current public health efforts to encourage the intake of fresh fruits and vegetables in the general population. Other general recommendations include limiting consumption of refined carbohydrates, sweets, processed and red meats.

## References

[1] Young RC (2002). Cancer statistics, 2002: Progress or cause for concern?. Ca-a Cancer Journal for Clinicians..

[2] Cartwright R, Brincker H, Carli PM, Clayden D, Coebergh JW, Jack A (1999). The rise in incidence of lymphomas in Europe 1985-1992. Eur J Cancer.

[3] Muller AMS, Ihorst G, Mertelsmann R, Engelhardt M (2005). Epidemiology of non-Hodgkin's lymphoma (NHL): trends, geographic distribution, and etiology. Annals of Hematology..

[4] Chow EJ, Holly EA (2002). Blood transfusions and non-Hodgkin's lymphoma. Epidemiol Rev..

[5] Zhang SM, Hunter DJ, Rosner BA, Giovannucci EL, Colditz GA, Speizer FE (2000). Intakes of fruits, vegetables, and related nutrients and the risk of non-Hodgkin's lymphoma among women. Cancer Epidemiol Biomarkers Prev.

[6] Kim YI (1999). Folate and cancer prevention: a new medical application of folate beyond hyperhomocysteinemia and neural tube defects. Nutr Rev.

[7] Oren T, Sher JA, Evans T (2003). Hematopoiesis and retinoids: development and disease. Leuk Lymphoma.

[8] Steinmetz KA, Potter JD (1991). Vegetables, fruit, and cancer. II. Mechanisms.Cancer Causes Control.

[9] Koutros S, Cross AJ, Sandler DP, Hoppin JA, Ma X, Zheng T (2008). Meat and meat mutagens and risk of prostate cancer in the Agricultural Health Study. Cancer Epidemiol Biomarkers Prev.

[10] Cross AJ, Ward MH, Schenk M, Kulldorff M, Cozen W (2006). Meat and meat-mutagen intake and risk of non-Hodgkin lymphoma: results from a NCI-SEER case-control study. Carcinogenesis.

[11] Harris NL, Jaffe ES, Diebold J, Flandrin G, Muller-Hermelink HK, Vardiman J, Lister TA, Bloomfield CD (1999). The World Health Organization classification of neoplastic diseases of the hematopoietic and lymphoid tissues. Report of the Clinical Advisory Committee meeting, Airlie House, Virginia, November, 1997. Ann Oncol.

[12] Mozaheb Z, Aledavood A, Farzad F (2011). Distributions of major sub-types of lymphoid malignancies among adults in Mashhad, Iran. Cancer Epidemiology..

[13] Boucher B, Cotterchio M, Kreiger N, Nadalin V, Block T, Block G (2006). Validity and reliability of the Block98 food-frequency questionnaire in a sample of Canadian women. Public health nutrition..

[14] Tsao JL, Dudley S, Kwok B, Nickel AE, Laird PW, Siegmund KD (2002). Diet, cancer and aging in DNA mismatch repair deficient mice. Carcinogenesis.

[15] Kelemen LE, Cerhan JR, Lim U, Davis S, Cozen W, Schenk M, Colt J, Hartge P, Ward MH (2006). Vegetables, fruit, and antioxidant-related nutrients and risk of non-Hodgkin lymphoma: a National Cancer Institute–Surveillance, Epidemiology, and End Results population-based case-control study. Am J Clin Nutr.

[16] Zhang S, Hunter DJ, Rosner BA, Colditz GA, Fuchs CS, Speizer FE (1999). Dietary fat and protein in relation to risk of non-Hodgkin's lymphoma among women. J Natl Cancer Inst.

[17] Skibola CF (2007). Obesity, diet and risk of non-Hodgkin lymphoma. Cancer Epidemiol Biomarkers Prev.

[18] Erber E, Maskarinec G, Gill JK, Park SY, Kolonel LN (2009). Dietary patterns and the risk of non-Hodgkin lymphoma: the Multiethnic Cohort. Leuk Lymphoma.

[19] Chiu BCH, Cerhan JR, Folsom AR, Sellers TA, Kushi LH, Wallace RB (1996). Diet and risk of non-Hodgkin lymphoma in older women. JAMA.

[20] De Stefani E, Fierro L, Barrios E, Ronco A (1998). Tobacco, alcohol, diet and risk of non-Hodgkin's lymphoma: a case-control study in Uruguay. Leukemia Research..

[21] Purdue MP, Bassani DG, Klar NS, Sloan M, Kreiger N (2004). Canadian Cancer Registries Epidemiology Research Group. Dietary factors and risk of non-Hodgkin lymphoma by histologic subtype: a case-control analysis. Cancer Epidemiol Biomarkers Prev.

[22] Kravchenko JS, Kris H (2008). Diet and Cancer. International Encyclopedia of Public Health.

[23] Calder PC, Kew S (2002). The immune system: a target for functional foods?. Br J Nutr.

[24] Zheng T, Holford TR, Leaderer B, Zhang Y, Zahm SH, Flynn S (2004). Diet and nutrient intakes and risk of non-Hodgkin's lymphoma in Connecticut women. Am J Epidemiol.

[25] Sauvaget C, Kasagi F, Waldren CA (2004). Dietary factors and cancer mortality among atomic-bomb survivors. Mutat Res.

[26] Han X, Zheng T, Foss F, Holford TR, Ma S, Zhao P (2010). Vegetable and fruit intake and non-Hodgkin lymphoma survival in Connecticut women. Leuk Lymphoma.

[27] Talamini R, Polesel J, Montella M, Dal Maso L, Crovatto M, Crispo A (2006). Food groups and risk of non-Hodgkin lymphoma: a multicenter, case-control study in Italy. Int J Cancer.

[28] Cross AJ, Lim U (2006). The role of dietary factors in the epidemiology of non-Hodgkin's lymphoma. Leuk Lymphoma.

[29] Zheng TZ, Holford TR, Leaderer B, Zhang YW, Zahm SH, Flynn S (2004). Diet and nutrient intakes and risk of non-Hodgkin's lymphoma in Connecticut women. American Journal of Epidemiology..

[30] Thompson CA, Habermann TM, Wang AH, Vierkant RA, Folsom AR, Ross JA (2010). Antioxidant intake from fruits, vegetables and other sources and risk of non-Hodgkin's lymphoma: the Iowa Women's Health Study. Int J Cancer.

[31] Chang ET, Smedby KE, Zhang SM, Hjalgrim H, Melbye M, Ost A (2005). Dietary factors and risk of non-hodgkin lymphoma in men and women. Cancer Epidemiol Biomarkers Prev.

[32] Chiu BC, Dave BJ, Ward MH, Fought AJ, Hou L, Jain S (2008). Dietary factors and risk of t(14;18)-defined subgroups of non-Hodgkin lymphoma. Cancer Causes Control.

[33] Thompson CA, Cerhan JR (2010). Fruit and vegetable intake and survival from non-Hodgkin lymphoma: does an apple a day keep the doctor away?. Leuk Lymphoma.

[34] Sedelnikova OA, Redon CE, Dickey JS, Nakamura AJ, Georgakilas AG, Bonner WM (2010). Role of oxidatively induced DNA lesions in human pathogenesis. Mutat Res.

[35] Franceschi S, Serraino D, Carbone A, Talamini R, La Vecchia C (1989). Dietary factors and non-Hodgkin's lymphoma: a case-control study in the northeastern part of Italy. Nutrition and cancer..

[36] Chao C, Page JH (2008). Type 2 diabetes mellitus and risk of non-Hodgkin lymphoma: a systematic review and meta-analysis. Am J Epidemiol.

[37] Ross M, Bras G (1965). Tumor incidence patterns and nutrition in the rat. Journal of Nutrition..

[38] Collins AR, Ferguson LR (2004). Nutrition and carcinogenesis. Mutat Res.

